# Prognostic factors and a new scoring system for survival of patients irradiated for bone metastases

**DOI:** 10.1186/s12885-019-6385-7

**Published:** 2019-11-28

**Authors:** Dirk Rades, Rapha Haus, Steven E. Schild, Stefan Janssen

**Affiliations:** 1 Department of Radiation Oncology, University of Lübeck, Ratzeburger Allee 160, D-23538 Lübeck, Germany; 2 Department of Radiation Oncology, Mayo Clinic Scottsdale, Scottsdale, AZ USA; 3 Medical Practice for Radiotherapy and Radiation Oncology, Hannover, Germany

**Keywords:** Bone metastases, Radiotherapy, Survival, Prognostic factors, Scoring system

## Abstract

**Background:**

Personalized therapy for bone metastases should consider the patients’ remaining lifespan. Estimation of survival can be facilitated with scoring tools. A new tool was developed, specifically designed to estimate 12-month survival.

**Methods:**

In 445 patients irradiated for bone metastases, radiotherapy regimen plus 13 factors (age, gender, Karnofsky performance score (KPS), primary tumor type, interval between cancer diagnosis and RT of bone metastases, visceral metastases, other (non-irradiated) bone metastases, sites of bone metastases, number of irradiated sites, pathological fracture, fractionation of RT, pre-RT surgery, pre-RT administration of bisphosphonates/denosumab, pre-RT systemic anticancer treatment) were retrospectively analyzed for survival. Factors achieving significance (*p* < 0.05) or borderline significance (*p* < 0.055) on multivariate analysis were used for the scoring system. Twelve-month survival rates were divided by 10 (factor scores); factor scores were summed for each patient (patient scores).

**Results:**

On multivariate analysis, survival was significantly associated with KPS (hazard ratio (HR) 1.91, *p* < 0.001) and primary tumor type (HR 1.12, p < 0.001); age achieved borderline significance (HR 1.14, *p* = 0.054). These factors were used for the scoring tool. Patient scores ranged from 8 to 17 points. Three groups were designated: 8–9 (A), 10–14 (B) and 15–17 (C) points. Twelve-month survival rates were 9, 38 and 72% (*p* < 0.001); median survival times were 3, 8 and 24 months.

**Conclusions:**

This new tool developed for patients irradiated for bone metastases at any site without spinal cord compression allows one to predict the survival of these patients and can aid physicians when assigning the treatment to individual patients.

## Background

The concept of personalized cancer care has become very popular during the last decade, particularly for patients with metastatic disease. The two most common metastatic sites treated by radiation oncologists are brain and bone. Bone metastases are quite common in adult cancer patients and occur in up to 70% of patients with breast or prostate cancer and up to 40% of patients with renal cell carcinoma during their malignant disease [1, 2]. Many of these patients receive radiotherapy (RT), either alone or, for example, in the case of an impending pathological fracture preceded by surgery. For radiotherapy of bone metastases, different dose-fractionation regimens are applied worldwide including single-fraction RT (e.g. 1 × 8 Gy), short-course multi-fraction regimens lasting for about 1 week (e.g. 5 × 4 Gy and 6 × 4 Gy) and longer-course regimens mostly lasting for 2 to 4 weeks (e.g. 10 × 3 Gy, 14–15 × 2.5 Gy and 18–20 × 2 Gy) [2]. Major goals of radiotherapy for bone metastases include relief of symptoms (mainly pain) and prevention of complications such as pathological fractures and spinal cord compression. Based on the results of several meta-analyses comparing single-fraction to multi-fraction RT, single-fraction RT is recommended for uncomplicated painful bone metastases, since single-fraction RT was similarly effective in decreasing pain as multi-fraction regimens [3–5]. However, re-irradiation was given significantly more often after single-fraction than after multi-fraction RT. Moreover, in a randomized trial recalcification of osteolytic bone was more pronounced after longer-course RT than after single-fraction RT [6]. The need for re-treatment and recalcification of osteolytic bone appear less important for patients with a short expected survival and single-fraction RT is considered their best option, since these patients likely do not live long enough to experience long term problems [2]. Thus, it is important to account for a patient’s remaining lifespan when personalizing treatment approach including the appropriate RT-regimen. Prognostic factors of survival and scoring instruments are very helpful for radiation oncologists in decision making. Estimation of a patient’s survival prognosis is of great value also for the patients themselves and their relatives when taking part in selection of treatment and planning their remaining life.

Very few scoring tools are currently available. One of the three most recent tools was developed from patients treated before 1999 and likely does not reflect the impact of modern targeted therapies on survival of cancer patients [7–9]. This aspect likely applies also to another most recently presented scoring tool, which was created from patients irradiated between 2001 and 2010 [10], and to a certain extent also to the third study including patients treated between 2000 and 2013 [11]. In the latter study, patients receiving orthopedic surgery without radiotherapy were included, which may have led to a treatment-related selection bias [11]. In the present study, all patients had received radiotherapy. Moreover, the patients were treated more recently than in these three prior studies, i.e. between 2009 and 2017. Additionally, different potential prognostic factors and dose-fractionation regimens were included in the present study compared to the previous studies. In contrast to one of the most recent previous scores [10], patients with metastatic spinal cord compression (MSCC) were excluded from the present study, since validated specific survival scores for MSCC already exist [12–14]. Moreover, patients with MSCC generally have worse survival prognoses than those patients with non-MSCC bone metastases and should be considered separately [2, 15]. Thus, the scoring instrument developed in this study can be considered supplementary to existing tools designed for estimating survival of patients irradiated for bone metastases.

## Methods

The data of 445 patients treated with fractionated RT for symptomatic (painful) bone metastases without spinal cord compression from a solid tumor at two German institutions between 2009 and 2017 were retrospectively analyzed. The study was approved by the ethics committee of the University of Lübeck and performed in accordance with the Helsinki Declaration. Since this is s retrospective study in a palliative situation, the majority of patients died prior to the collection of the data for this study. Moreover, the data used for the study were anonymized after completion of the data entry. Therefore, the ethcics comittee approved the study without requesting written informed consent to participate from the patients. Criteria for inclusion in this study included evidence of bone metastases requiring palliative RT and confirmation of bone metastases by magnetic resonance imaging or computed tomography (CT). Patients with hematologic malignancies were excluded. Radiotherapy was performed with 6–18 MV photon beams from a linear accelerator after CT-based treatment planning. In Germany, the vast majority of patients with bone metastases are treated with fractionated RT, and single-fraction RT is generally limited to few patients with an extraordinarily poor estimated survival. Since fractionated RT is the standard treatment for bone metastases in the centers participating in this study and single-fraction RT represents a major exception, patients receiving single-fraction RT were not included in the present study to reduce the risk of a potential selection bias. The fractionation-regimen of RT (short-course with 5–6 × 4 Gy vs. longer-course RT with 30–40 Gy in 10–20 fractions, mainly (86%) 10 × 3 Gy and 14–15 × 2.5 Gy) plus 13 pre-RT factors were investigated for potential associations with survival. These factors included age at the time of RT (≤60 vs. 61–70 vs. > 70 years), gender, Karnofsky performance score (KPS) (≤70 vs. 80–100), primary tumor type (*N* ≥ 20) (breast cancer vs. prostate cancer vs. lung cancer vs. renal cell carcinoma vs. colorectal cancer vs. other tumors), interval between cancer diagnosis and RT of bone metastases (≤8 vs. ≥9 months, median interval = 9 months, visceral metastases (no vs. yes), other (non-irradiated) bone metastases (no vs. yes), location of irradiated bone metastases (spinal site(s) only vs. extraspinal site(s) with or without spinal site(s)), number of irradiated sites (single vs. multiple), pathological fracture (no vs. yes), pre-RT surgery (no vs. yes), pre-RT administration of bisphosphonates/denosumab (no vs. yes), and pre-RT systemic anticancer treatment (no vs. yes). The distributions of these factors are shown in Table 1. The type of pre-RT systemic treatment (immunotherapy, chemotherapy, targeted therapy) was not considered as a separate factor, since this would apply to only 71% of the patients (Table 1). Moreover, since the type of systemic treatment is closely related to the type of primary tumor, tumor type and type of systemic treatment will likely be confounding variables. Comorbidity scores such as the Charlson comorbidity index or the age-adjusted Charlson comorbidity index were not considered, since these scores are generally dominated by the presence of a metastatic solid tumor, which applies to all patients of the present study [16, 17]. Moreover, since a high comorbidity index is usually associated with a poor performance score, comorbidity score and performance score will likely be confounding variables.

**Table 1:** Distributions of the factors investigated for potential associations with survival

	N patients (%)
Age	
≤ 60 years	132 (30)
61–70 years	131 (29)
> 70 years	182 (41)
Gender	
Female	226 (51)
Male	219 (49)
Karnofsky performance score	
≤ 70	165 (37)
80–100	237 (53)
Unknown	43 (10)
Type of primary tumor	
Breast cancer	125 (28)
Prostate cancer	59 (13)
Lung cancer	138 (31)
Renal cell carcinoma	24 (5)
Colorectal cancer	20 (4)
Other tumors	79 (18)
Interval between cancer diagnosis and RT of bone metastases	
≤ 8 months	226 (51)
≥ 9 months	219 (49)
Visceral metastases at the time of RT	
No	187 (42)
Yes	258 (58)
Other (non-irradiated) bone metastases at the time of RT	
No	109 (24)
Yes	334 (75)
Unknown	2 (< 1)
Location of irradiated bone metastases	
Spinal site(s) only	170 (38)
Extraspinal site(s) with or without spinal site(s)	275 (62)
Number of irradiated sites	
Single	186 (42)
Multiple	259 (58)
Pathological fracture	
No	309 (69)
Yes	136 (31)
Pre-RT surgery (no vs. yes)	
No	347 (78)
Yes	96 (22)
Unknown	2 (< 1)
Pre-RT administration of bisphosphonates/denosumab	
No	268 (60)
Yes	167 (38)
Unknown	10 (2)
Pre-RT systemic anticancer treatment	
No	127 (29)
Yes	317 (71)
Unknown	1 (< 1)
Fractionation of RT	
Short-course RT	43 (10)
Longer-course RT	402 (90)

Survival time was referenced from the first day of RT. Survival rates were calculated with the Kaplan-Meier-method, and differences between the Kaplan-Meier curves were calculated with the log-rank test (univariate analyses). Those factors that achieved significance (*p* < 0.05) or borderline significance (*p* < 0.055) on univariate analysis were included in a multivariate analysis performed with the Cox proportional hazards model. Factors achieving significance (p < 0.05) or borderline significance (p < 0.055) on multivariate analysis were used to generate the scoring system.

## Results

On univariate analyses, improved survival was significantly associated with female gender (*p* < 0.001), KPS 80–100 (p < 0.001), more favorable primary tumor type (breast cancer, prostate cancer, renal cell carcinoma) (p < 0.001), longer interval (≥9 months) between cancer diagnosis and RT of bone metastases (*p* = 0.044), absence of visceral metastases (*p* = 0.002), absence of a pathological fracture (*p* = 0.049) and longer-course RT (*p* = 0.022). The complete results (6-month and 12-month survival rates) of the univariate analyses are shown in Table 2.

**Table 2:** Univariate analyses of survival

	Survival rate at 6 months (%)	Survival rate at 12 months (%)	*p*-value
Age			
≤ 60 years	61	48	
61–70 years	60	44	
> 70 years	60	38	0.052
Gender			
Female	64	50	
Male	56	35	**< 0.001**
Karnofsky performance score			
≤ 70	42	23	
80–100	70	55	**< 0.001**
Type of primary tumor			
Breast cancer	78	64	
Prostate cancer	78	52	
Lung cancer	46	30	
Renal cell carcinoma	79	63	
Colorectal cancer	45	25	**< 0.001**
Other tumors	42	22	
Interval between cancer diagnosis and RT of bone metastases			
≤ 8 months	54	40	
≥ 9 months	66	45	**0.044**
Visceral metastases at the time of RT			
No	67	49	
Yes	55	38	**0.002**
Other (non-irradiated) bone metastases at the time of RT			
No	53	36	
Yes	63	45	0.23
Location of irradiated bone metastases			
Spinal site(s) only	59	40	
Extraspinal site(s) with or without spinal site(s)	61	44	0.95
Number of irradiated sites			
Single	59	43	
Multiple	61	42	0.41
Pathological fracture			
No	61	46	
Yes	57	35	**0.049**
Pre-RT surgery (no vs. yes)			
No	57	42	
Yes	72	48	0.17
Pre-RT administration of bisphosphonates/denosumab			
No	56	41	
Yes	65	45	0.55
Pre-RT systemic anticancer treatment			
No	55	40	
Yes	62	44	0.33
Fractionation of RT			
Short-course RT	40	26	
Longer-course RT	62	45	**0.022**

Bold values = significant *p*-values

**Fig. 1:**
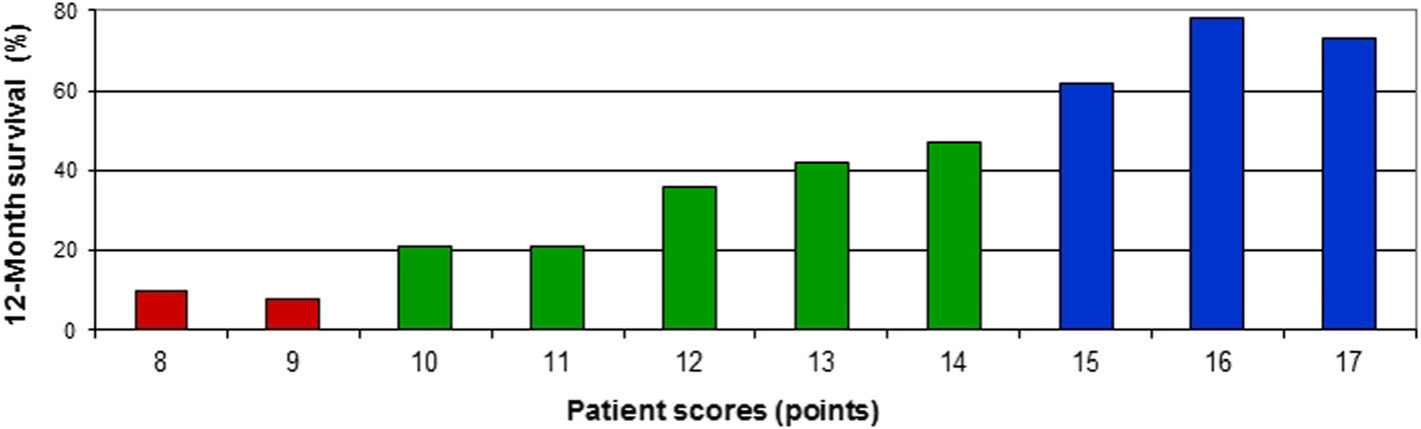
Patient scores related to the corresponding 12-month survival rates

**Fig. 2:**
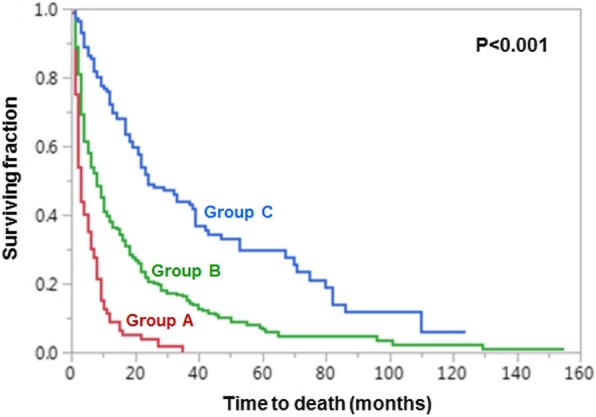
Kaplan-Meier curves of the three prognostic groups A (8–9 points), B (10–14 points) and C (15–17 points). The *p*-value was calculated with the log-rank test

On multivariate analysis (Cox proportional hazards model, Table 3), survival was significantly associated with KPS (hazard ratio (HR) 1.91, 95%-confidence interval (CI) 1.51–2.41, *p* < 0.001) and primary tumor type (HR 1.12, 95%-CI 1.07–1.17, p < 0.001). Age achieved borderline significance (HR 1.14, 95%-CI 1.00–1.30, *p* = 0.054). Therefore, these three factors were used for creating the scoring tool. Since the KPS was not known in 43 patients (9.7%), the scoring system was built using the data of 402 patients. The 12-month survival rates of each significant factor (in percent) were divided by 10 to obtain the factor scores (Table 4). The factor scores were added for each patient, and the patient scores were received that ranged from 8 to 17 points (Fig. 1). Based on the patient scores, three prognostic groups were designated: 8–9 points (group A), 10–14 points (group B) and 15–17 points (group C). The 12-month survival rates of these groups were 9, 38 and 72%, respectively (Fig. 2, *p* < 0.001). The 6-month survival rates were 30, 54 and 85%, respectively, and the median survival times were 3 months, 8 months and 24 months, respectively (p < 0.001).

**Table 3:** Multivariate analyses of survival (Cox proportional hazards model)

	Hazard ratio	95% confidence interval	*p*-value
Age	1.14	1.00–1.3	0.054
Gender	1.20	0.95–1.51	0.13
Karnofsky performance score	1.91	1.51–2.41	**< 0.001**
Type of primary tumor	1.12	1.07–1.17	**< 0.001**
Interval between cancer diagnosis and RT of bone metastases	1.14	0.92–1.42	0.23
Visceral metastases at the time of RT	1.19	0.96–1.49	0.12
Pathological fracture	1.19	0.93–1.51	0.16
Fractionation of RT	1.02	0.86–1.22	0.84

Bold values = significant *p*-values

**Table 4:** Survival rates at 12 months of the prognostic factors found significant or almost significant on multivariate analysis and the corresponding factor scores

Prognostic factor	Survival rate at 12 months (%)	Factor score
Age		
≤ 60 years	48	5
61–70 years	44	4
> 70 years	38	4
Karnofsky performance score		
≤ 70	23	2
80–100	55	6
Type of primary tumor		
Breast cancer	64	6
Prostate cancer	52	5
Lung cancer	30	3
Renal cell carcinoma	63	6
Colorectal cancer	25	3
Other tumors	22	2

## Discussion

Bone metastases are common in cancer patients with advanced disease. Radiotherapy, either alone or following orthopedic surgery, is the most frequently used treatment to control symptoms and prevent complications. In order to provide a treatment that is optimally tailored to a patient’s individual situation, it is important to estimate his remaining lifespan as precisely as possible. This can be facilitated with prognostic tools. Some survival scores specifically designed for patients irradiated for bone metastases are available. Most scores are relatively old and likely do not consider the potential impact of modern targeted therapies on survival of these patients [11, 12].

In 2005, scoring tool was developed from 342 patients of the Dutch Bone Metastasis Study who were irradiated with 1 × 8 Gy or 6 × 4 Gy for spinal metastases without major neurologic impairment and vertebral collapse between 1996 and 1998 [18]. Based on three independent significant predictors, i.e. KPS (*p* < 0.001), primary tumor type (p < 0.001) and visceral metastases (*p* = 0.02), three prognostic groups were formed with median survival times of 3.0, 9.0 and 18.7 months, respectively. For several years, this was the only well-recognized survival score developed in patients irradiated for bone metastases. However, in contrast to our present study, it was limited to metastases of the spine and did not consider other sites such as pelvis, ribs, shoulders, extremities and skull. Therefore, the prognostic groups identified in that study may not applicable to patients with metastatic sites other than the spinal column [18]. In 2014, two additional survival scores for patients receiving radiotherapy for bone metastases were presented [7, 10]. One score was also developed from patients treated within the Dutch Bone Metastasis Study with 1 × 8 Gy or 6 × 4 Gy, this time from the entire cohort of 1157 patients [7]. Several independent predictors of survival were identified including gender, primary tumor type, KPS, visceral metastases and two self-rating scores for patients regarding general health and overall valuation of life. Since this model appeared very complex, the authors added a simpler tool including only KPS and primary tumor type. They stated the 3 to 18 months survival rates for breast cancer, prostate cancer, lung cancer and other cancer patients related to three KPS levels (90–100, 70–80 and 20–60) but did not create prognostic groups that are usually part of a scoring instrument. The second tool published in 2014 included the retrospective data of 1043 patients treated for spinal metastases between 2001 and 2010 [10]. Ninety-nine percent of the patients received RT with or without surgery that was mainly delivered as single-fraction RT with 1 × 8 Gy (43%) or short-course RT with 2 × 8 Gy (16%) or 5–6 × 4 Gy (31%). Four categories were designated based on KPS (80–100 vs. 10–70) and visceral/brain metastases (no vs. yes) with median survival times of 31.2, 15.4, 4.8 and 1.6 months, respectively. Also this score may not be applicable to patients with bone metastases at sites other than spinal column. In contrast to the other survival scores for patients irradiated for bone metastases, 49% of the patient used for this tool had symptomatic MSCC with motor and/or sensory deficits [10]. Due to the fact that in general patients with MSCC have worse survival prognoses than patients with painful bone metastases and no MSCC [2, 15], we believed that these cohorts should not be mixed for the development of a survival score and that separate tools for patients with bone metastases with and without MSCC should be provided to facilitate optimal personalization of the treatment. The most recent survival score for patients irradiated for bone metastases was presented in 2018 and was created from the data of 1520 patients treated for symptomatic metastases of the long bones between 2000 and 2013 [11]. Based on three independent prognostic factors, clinical profile, KPS and visceral/brain metastases, four categories were designed with median survival times of 21.9, 10.5, 4.6 and 2.2 months, respectively. Since this tool was developed from patients with long bone metastases only, its applicability may not be generalized to patients with bone metastases at other sites. Moreover, the fact that 130 patients (8.6%) received surgery alone without radiotherapy may have led to a selection bias during the development of the scoring tool.

Considering the existing scoring instruments, we feel that an additional tool for patients irradiated for bone metastases that considers all sites but excludes patients with MSCC and those not receiving radiotherapy would be particularly useful. The present study was performed to close this gap. The new tool was specifically designed to predict the 12-month survival probability of patients receiving radiotherapy for bone metastases but can also be used to estimate the 6-month survival. When using this tool, the retrospective nature of the data needs to be considered. Retrospective studies bear the risk of hidden selection biases. To reduce the risk of a fractionation-related bias, only patients treated with fractionated RT were included. Fractionated RT is the standard treatment for bone metastases in the centers contributing to this study, whereas single-fraction RT is used only for a small minority of patients with very limited survival times. In the present study, longer-course RT was associated with a better survival than short-course multi-fraction RT on univariate analysis (Table 2). Although no significant association between the fractionation regimen and survival was found on multivariate analysis (Table 3), a selection bias caused by the fact that longer-course RT was applied more frequently to patients with longer expected survival cannot be completely excluded. Another potential limitation of the study is the fact that the performance score was unknown in 43 patients (9.7%).

For selection of the appropriate dose-fractionation regimen for an individual, one should keep in mind that in a randomized trial, recalcification of the osteolytic bone was more pronounced after longer-course RT with 10 × 3 Gy than after single-fraction RT with 1 × 8 Gy, which is particularly important for patients with favorable survival prognoses [9]. In the present study, patients of group A had the worst prognoses with a median survival time of only 3 months and 6- and 12-month survival rates of only 30 and 9%, respectively. Therefore, the majority of these patients will likely not live long enough to require re-irradiation for bone pain and, therefore, appear good candidates for single-fraction RT. Also patients with very limited survival prognoses were reported to benefit from RT in terms of symptom relief [19, 20]. According to several studies, single-fraction RT with 8 Gy was superior to 4 Gy with respect to pain relief and should be the preferred dose for single-fraction RT of painful bone metastases [21–24]. For patients of group B who had a median survival time of 8 months and an intermediate 12-month survival probability of 38%, single-fraction RT with 1 × 8 Gy and short-course multi-fraction RT with 5–6 × 4 Gy may be considered, depending on patient preference. If recurrent pain occurs after single-fraction RT, a second course of single-fraction RT can be safely administered [25]. However, about 40% of the patients do no benefit from re-irradiation [26]. Patients of group C had very favorable survival prognoses with a median survival time of 24 months and a 12-month survival probability of 72%. Thus, many of these patients will likely live long enough to experience recurrent bone pain in irradiated sites after single-fraction RT or short-course RT. Therefore, these patients appear more suitable for longer-course RT with total doses of 30–40 Gy and doses per fraction of ≤3 Gy. Particularly patients of group C with spinal metastases may be candidates for stereotactic body RT (SBRT) rather than conventional RT, since SBRT can result in considerable long-term control of pain [27]. When following these suggestions, the retrospective nature of the present study should be considered. Moreover, the results may not be generalized to patients treated with single-fraction RT or SBRT. Since the predictive factors used for the scoring tool include the primary tumor type, estimation of a patient’s prognosis may be made by experienced (radiation) oncologists, who are specialized in the treatment of a specific tumor entity, without using this new tool.

## Conclusion

This new tool allows predict the survival prognoses of patients to be irradiated for bone metastases without MSCC and allow physicians to choose the best treatment for an individual patient. It was designed to be used for bone metastases at any site. Since visceral metastases were not included in this score, a complete staging of the malignant disease is not required, which facilitates the use of this tool. External validation is planned to be performed in Europe and North America within the next years.

## Data Availability

The data analyzed for this paper cannot be shared on a publicly available repository due to data protection regulations. According to the local ethics committee, only the evaluation of anonymized data is allowed for this study.

## Abbreviations

CI: Confidence interval
CT: Computed tomography
HR: Hazard ratio
KPS: Karnofsky performance score
MSCC: Metastatic spinal cord compression
RT: Radiotherapy
SBRT: Stereotactic body radiation therapy
